# Using a Dynamic Hydrology Model To Predict Mosquito Abundances in Flood and Swamp Water

**DOI:** 10.3201/eid0801.010049

**Published:** 2002-01

**Authors:** Jeffrey Shaman, Marc Stieglitz, Colin Stark, Sylvie Le Blancq, Mark Cane

**Affiliations:** Columbia University, Palisades, New York, USA

**Keywords:** entomology, hydrology, Culicidae, *Aedes*, *Anopheles*, *Culex*, vector control, *West Nile virus*, St. Louis encephalitis, dynamic modeling

## Abstract

We modeled surface wetness at high resolution, using a dynamic hydrology model, to predict flood and swamp water mosquito abundances. Historical meteorologic data, as well as topographic, soil, and vegetation data, were used to model surface wetness and identify potential fresh and swamp water breeding habitats in two northern New Jersey watersheds. Surface wetness was positively associated with the subsequent abundance of the dominant floodwater mosquito species, *Aedes vexans,* and the swamp water species, *Anopheles walkeri.* The subsequent abundance of *Culex pipiens,* a species that breeds in polluted, eutrophic waters, was negatively correlated with local modeled surface wetness. These associations permit real-time monitoring and forecasting of these floodwater and nonfloodwater species at high spatial and temporal resolution. These predictions will enable public health agencies to institute control measures before the mosquitoes emerge as adults, when their role as transmitters of disease comes into play.

In their efforts to control mosquitoes and mosquito-borne diseases, public health officials would benefit if they could identify the locations of mosquito populations. Unfortunately, most public health agencies lack the resources for comprehensive sampling and monitoring of the spatial and temporal distribution of mosquito populations. In an effort to circumvent this shortcoming, researchers have attempted to account for fluctuations in mosquito populations through the monitoring of environmental conditions. Many such studies have associated the measured abundance of vectors or vector-borne disease incidence with satellite imaging (*1*–*8*). Such studies have generally used vegetation classification or the normalized differential vegetation index, which measures vegetation greenness, as proxies for soil moisture.

Patz et al. (*9*) estimated soil moisture more directly by using a dynamic hydrology model. Those researchers used a water balance model to hindcast weekly soil moisture levels in the Lake Victoria basin. (Hindcast is the retrospective prediction of historical conditions.) These soil moisture levels were then associated with local mosquito biting rates on humans and entomologic inoculation rates. This study demonstrated the potential application of dynamic hydrology models in epidemiologic monitoring; however, the model was coarse in both temporal and spatial resolution and lacked the means for assessing the spatial distribution of wetness across the land surface.

We present an example of how flood and swamp water mosquito abundance can be predicted in real time at high spatial resolution through application of a more detailed dynamic hydrology model (*10*). This model accounts for topographic variability and its control over soil moisture heterogeneity and runoff within a watershed. In doing so, the model resolves small areas of surface wetness and permits identification of the spatial distribution of potential breeding habitats within a catchment.

Our approach consisted of two components: physical and empirical. First, we used the dynamic hydrology model to hindcast the surface wetness (puddles, bogs, ponds) that potentially support floodwater and swamp water mosquito larvae. Then the spatial-temporal variability of this model-predicted surface wetness was empirically associated with the spatial-temporal variability of floodwater and swamp water mosquito abundances. We would have preferred to make this empirical association directly with larval collection data; however, because such data were not available, we established this association with adult mosquitoes collected in light traps. The result of this dynamic-empirical analysis is a logistic regression model fit relating local surface wetness to subsequent mosquito species abundances.

## Modeling Overview and Methods

Surface wetness conditions are a function not only of precipitation and temperature but also of a number of other meteorologic variables controlling evaporation, as well as soil properties, vegetation, and antecedent conditions (prior wetness in the watershed). In addition, topography controls much of the spatial distribution of wetness in a watershed, i.e., valleys are generally wetter than the drier upland regions. Our hydrology model incorporates all these factors, using topography to account for the distribution of wetness across the land surface (*10*). The model tracks the expansion and contraction of lowland saturated zones within this spatial framework and generates a picture of the variable wetness of the land surface as it changes in time.

**Figure 1:**
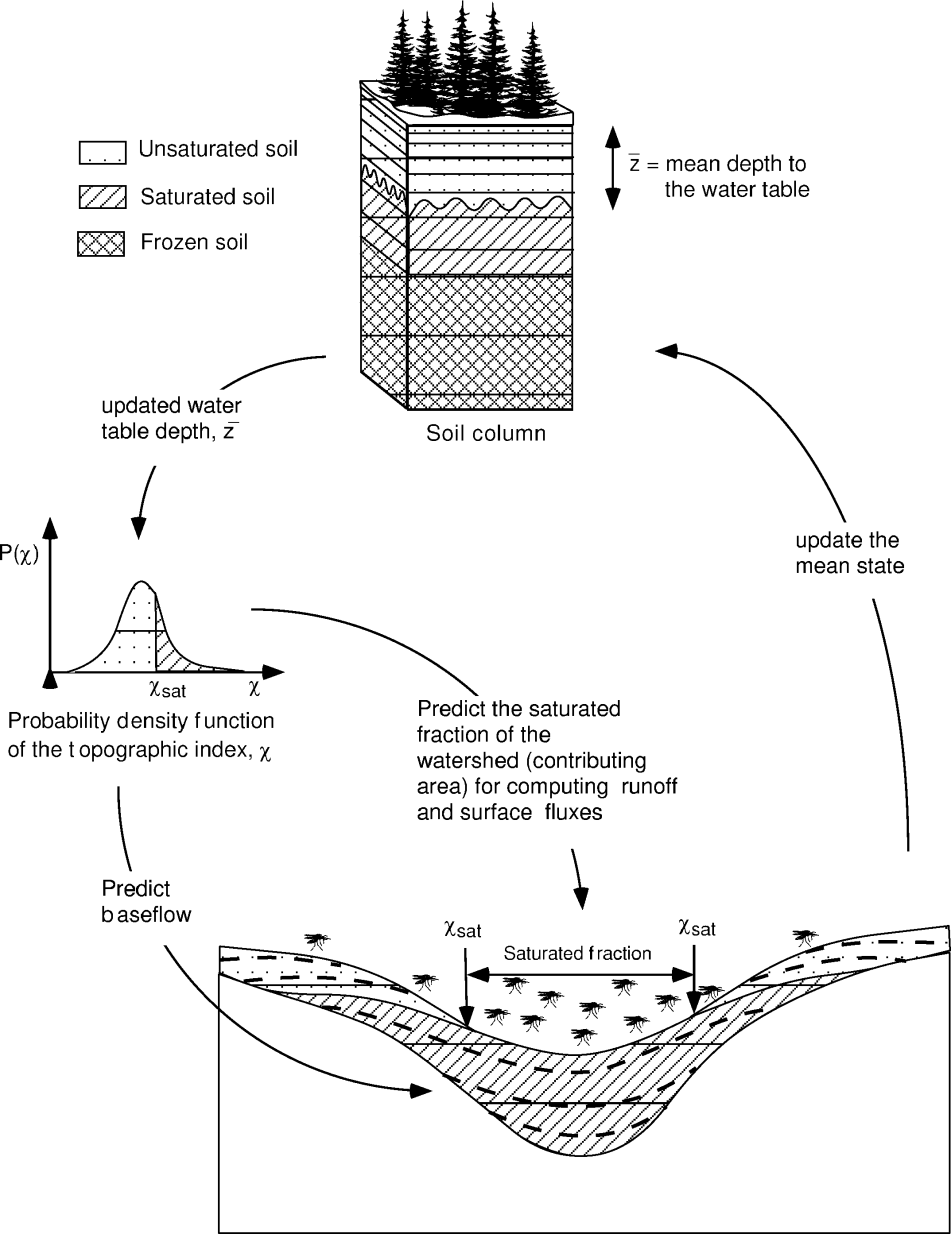
Visualization of the processing of land surface topography for a sample 25-km^2^ area in New York State. a) Digital Elevation Model--a pixelated (10 m cells) representation of land surface topography. Contour lines (in meters) have been overlain. b) Postprocessing, depiction of land surface wetness at a single point in time. Blue areas are wettest. The variability and spatial distribution of surface wetness are evident.

**Figure 2:**
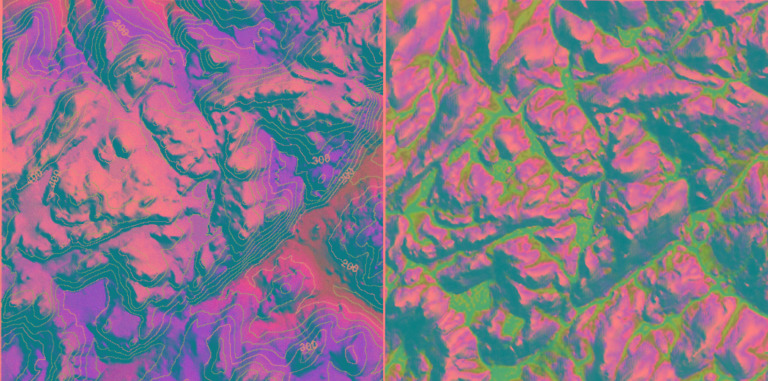
Schematic depiction of the hydrology model. The model couples the analytic form of TOPMODEL equations within a single column framework. From an update of the mean water table depth, TOPMODEL equations and Digital Elevation Model data are used to generate baseflow and the saturated fraction of the watershed.

For both large and small watersheds, it is possible to model surface wetness across individual plots of land as small as 100 m^2^ (e.g., 10-m cells) and monitor how the surface wetness of these plots changes through time. We use an adaptation of TOPMODEL, a conceptual framework for rainfall-runoff modeling (*11*–*14*). Our model uses gridded digital maps of land surface topography and a dynamic numerical framework that accounts for the movements of water and energy within the soil and at the surface. The mean depth of the water table and the statistics of topography within a watershed are used to compute the saturated areas of the watershed and the shallow groundwater flow that supports it (Figure 1). Thus, at any point in time we can create a mosaic of cells, each with a local model surface wetness, which taken as a whole represents the surface conditions of the entire watershed (Figure 2). This mosaic of surface wetness depicts the spatial variability of conditions at the land surface that result from terrain (topography, vegetation, and soil type) and integrated weather forcing (meteorologic conditions).

The modeling framework can be run continuously and has been validated at catchments ranging in scale from the Red Arkansas basin (570,000 km^2^) (*15*) to the Imnavait Creek catchment (2.2 km^2^) (*16*). Real-time surface wetness conditions can be generated by using current weather conditions. In addition, the hydrology model can be assimilated within the framework of global climate models, allowing integrated medium- to long-term forecasts of surface wetness conditions at a similar spatial resolution.

## Application and Validation of the Hydrology Model

The probability distribution of the soil moisture deficit, i.e., statistics of topography, is generated from digital elevation model data by using a multidirectional flow routing algorithm, which is tied to an adaptive error correction (pit infill) scheme needed for low-relief areas such as coastal plains. Our algorithm is similar to that of Martz and Garbrecht (*17*).

The hydrology model is driven by an hourly record of air temperature, precipitation, relative humidity, surface pressure, wind speed, incident long-wave radiation, and solar radiation. Model simulation yields both hourly and daily time series of several catchment hydrologic variables, including mean water table depth (WTD), percent surface saturation, and total surface runoff. The last variable is used to generate a hydrograph for the outlet river of the catchment, which then can be compared with actual weir measurements.

### Linking the Model to Mosquitoes

From the mean catchment WTD, a time series of local WTD can be calculated for any pixel within the watershed. Thus, the hydrology model links weather to changes in local surface wetness, including the saturated surface regions (i.e., pools and swamps) exploited by floodwater mosquitoes. The development time of these mosquitoes from egg until emergence as adult is 7 to 20 days, depending on species and temperature conditions (*18*,*19*). Because the model provides a time series of hydrologic variables affecting surface wetness at hourly and daily intervals, it captures the expansion and contraction of saturated surface regions (i.e., breeding pools) at rates that impact mosquito development.

Monitoring local WTDs also permits detection of mosquito breeding pools at very fine, subpixel, spatial scales. A modeled, local WTD for a given pixel of -0.2 m does not imply that the whole of the 30-m x 30-m pixel is dry, merely that the pixel mean elevation is. Given the variability in surface elevation--and in the water table depth--a percentage of the water table can be expected to outcrop. The shallower the local WTD for a given pixel, the greater the percentage of that pixel area that can be estimated to be wet at the surface. Thus, a pixel with a WTD of -0.4 m can be expected to have more surface pooling than a pixel with a WTD of -1.4 m. Both models and data have shown that substantial soil moisture heterogeneity exists at most scales within a catchment (*20*,*21*). This fractal geometry permits such extrapolation of the pixel-to-pixel variability of local WTDs to a smaller, subpixel scale. Hoof prints, ditches, tire tracks, and natural relief can all account for the heterogeneity of elevation within a pixel. Use of local WTD allows a statistical estimation of such potentially saturated portions of the surface.

## Data Collection and Analysis

**Figure 3:**
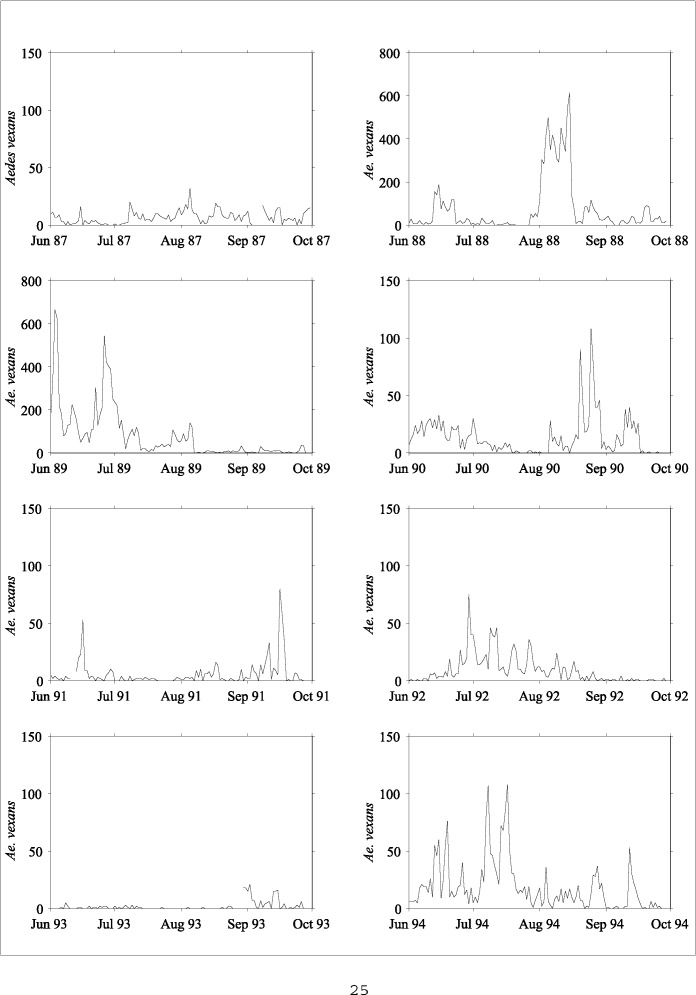
*Aedes vexans* collections at the Great Swamp, 1987 - 1994.^a a^Considerable year-to-year variability is evident in these light trap collections. Note the different scaling for 1988 and 1989.

Two adult mosquito collection records from northern New Jersey were used: 1) a 13-year time series of daily mosquito counts (May through September) from seven sites near the Pequest River, and 2) a 15-year time series of daily mosquito counts (June through September) from a single site in the Great Swamp National Wildlife Refuge. (Collections at the Pequest River were not made every year at all seven sites; instead, records at these individual sites are from 1 year to 10 years of the full 13-year record.) Collections at each site were made with a New Jersey light trap and were counted and sorted by species. Trap locations were identified with a global positioning sensor. Figure 3 shows a representative 8-year time series of the floodwater species *Ae. vexans,* collected at the Great Swamp site.

Hourly meteorologic data were assembled from National Climate Data Center (NCDC) archives for nearby Allentown, Pennsylvania. Solar radiation data were provided by the Northeast Regional Climate Center (NRCC) from analysis of the NCDC data by the NRCC solar energy model (*22*). Catchment topographic statistics were generated from 30-m pixel resolution DEMs delineating the Pequest River and Great Swamp watersheds downloaded from the U.S. Geological Survey (USGS) website (*23*). The hydrology model was validated against USGS hydrograph data for the Pequest River catchment.

Two time series of daily mean catchment WTD were generated--one for the Pequest River catchment and one for the Great Swamp catchment. WTDs were then used to create indices of local wetness (ILWs), time series measures of the surface wetness of the immediate area surrounding each of the seven mosquito collection sites in the Pequest River catchment and the one site in the Great Swamp catchment. At each mosquito collection site, an ILW was constructed by using a square block of pixels--24 pixels on a side, centered about the geocoordinates of the site. Instead of elevation, each pixel was represented by its soil moisture deficit value. Using this square grid of pixels and the daily time series of mean catchment WTD, we computed the local WTD for each pixel at each daily time step. A threshold depth was then chosen (e.g., 0 m or 0.5 m; analyses were insensitive to this range of depth values). Every pixel in the matrix was then assigned a new value: one if the WTD for the pixel were higher than the chosen depth value or zero if the WTD for the pixel were deeper than the chosen depth. This created a binary 24-by-24 matrix of ones and zeros at each daily time step. These numbers were summed for each time step to create an ILW, a time series array of values representing the local wetness of a collection site.

Statistical analyses were then used to estimate the association between modeled surface wetness--i.e., the ILW and mosquito species prevalence. Time series regression analyses were used at each site to determine the tendency and strength of the association between mosquito abundances and the collocated ILW. The time series regressions were performed by using the log (mosquitoes + 1), which normalized the collection data. Lag correlations were adopted to represent the average time of mosquito development from ovipositing until emergence.

From a public health perspective, it is often the mass emergence of mosquitoes that criticially needs to be identified. We used logistic regression to explore the ability of modeled surface wetness (the ILW) to predict the timing of such events. Logistic regression analyses were used to estimate the probability of a mass emergence, which we defined as a single-day collection of either ≥128, or ≥32 mosquitoes of a given species, depending on collection abundance. Multiple logistic regression analysis was used to determine the probabilities that collected mosquitoes would exceed a range of values (powers of two).

Analyses at the Pequest River sites were confounded by mosquito control efforts--spraying of both larvicide and adulticide in the areas of mosquito collection. As a consequence, regression analysis was restricted to the dominant floodwater species of the area, *Ae. vexans*. Logistic regression analyses were performed only at the Great Swamp site--the area without mosquito control.

## Results

**Figure 4:**
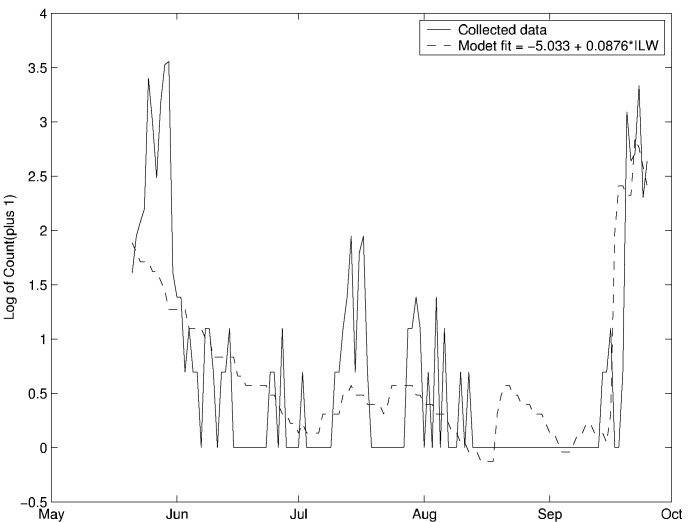
Time-series regression model fit of *Aedes vexans* 10 days later at the Bernaski site, Pequest River catchment. Regression fit is significant at p<0.01, r-squared = .50.

Table 1 presents correlation coefficients based on time- series regression analyses of *Ae. vexans* at two Pequest River catchment sites: Bernaski, for which there was 1 year of mosquito collection data; and Youngs Island, for which there were 10 years of collection data. At the Bernaski site, 50% of the variance in adult *Ae. vexans* was explained by the ILW (r = 0.70, p <0.01) with a 10-day lag (Table 1). Ten days is approximately the mean time of development of *Ae. vexans* from egg until emergence as adult, the period in which pooled surface habitats are necessary for mosquito survival (*24*). Figure 4 shows the regression model fit for this site. At the Youngs Island site, there is notable year-to-year variability in the correlation of the ILW with the abundance of *Ae. vexans* 10 days subsequent (Table 1). 1987 is the only year for which the association is positive and statistically significant (p<0.01). 1987 was also the only year without mosquito control at the Youngs Island site.

**Table 1:** Yearly correlation coefficients, two Pequest River sites^a^

Year	Correlation coefficient (r)	Sample size (n)
Bernaski site		
1987	0.7046^b^	128
Youngs Island site		
1987	0.3748^b^	134
1988	0.1497	137
1990	0.0090	138
1991	-0.1038	151
1993	0.1271	150
1994	-0.4666^b^	147
1995	-0.2294^c^	130
1996	0.0474	153
1997	0.0431	153
1998	0.0256	147

^a^Correlation coefficients based on yearly regression analyses of *Ae. vexans* at two sites in the Pequest River catchment: a) Bernaski and b) Youngs Island. The index of local wetness was constructed for a depth of 0.5 m. log (count + 1), lagged 10 days, and used in the regression analyses.

^b^p<0.01.

^c^p<0.05.

No mosquito control took place at the Great Swamp, and light trap collections were greatest at this site. Table 2 presents correlation coefficients based on time-series regression analyses of the three most abundant mosquito species. For the full 15-year record, the ILW was positively correlated with a 10-day lag (p<0.0001) to both *Ae. vexans* and *An. walkeri*, a swamp water species (*25*). The ILW was negatively correlated with a 10-day lag (p<0.0001) to *Cx. pipiens*, a species that breeds in more polluted, eutrophic waters (*26*). While these values are statistically significant, the ILW fails to explain >12% of the variance of any of the mosquito species analyzed.

**Table 2:** Yearly correlation coefficients (r) Great Swamp site.10-day lag, log (count + 1) (p<0.05)^a^

Year	*Aedes vexans*	*Anopheles walkeri*	*Culex pipiens*
All 15 years	0.3433	0.2693	-0.2623
1984	0.5705	-	-
1985	0.3023	-	-
1986	-0.4186	-	-0.5698
1987	-	-	-0.2695
1988	0.2479	0.3380	-0.2888
1989	0.8018	0.4551	-0.6007
1990	0.5505	-	-0.2507
1991	-	-	-0.4271
1992	0.2022	-	-
1993	-	0.4502	-0.6108
1994	-0.4699	-0.7101	-0.6099
1995	0.5453	0.4047	0.2657
1996	0.5927	0.3445	0.2914
1997	-	-	-0.2172
1998	0.6376	0.5341	-

^a^Correlation coefficients based on full-record and yearly time-series regression analyses of the three most abundant species collected at the Great Swamp. The ILW was constructed for a depth of 0 m. log (count + 1), lagged 10 days, and used in the time-series regression analyses. Correlation coefficients for the full 15-year record are significant (p<0.0001). Yearly correlation coefficient values are listed only if significant at p<0.05.

**Figure 5:**
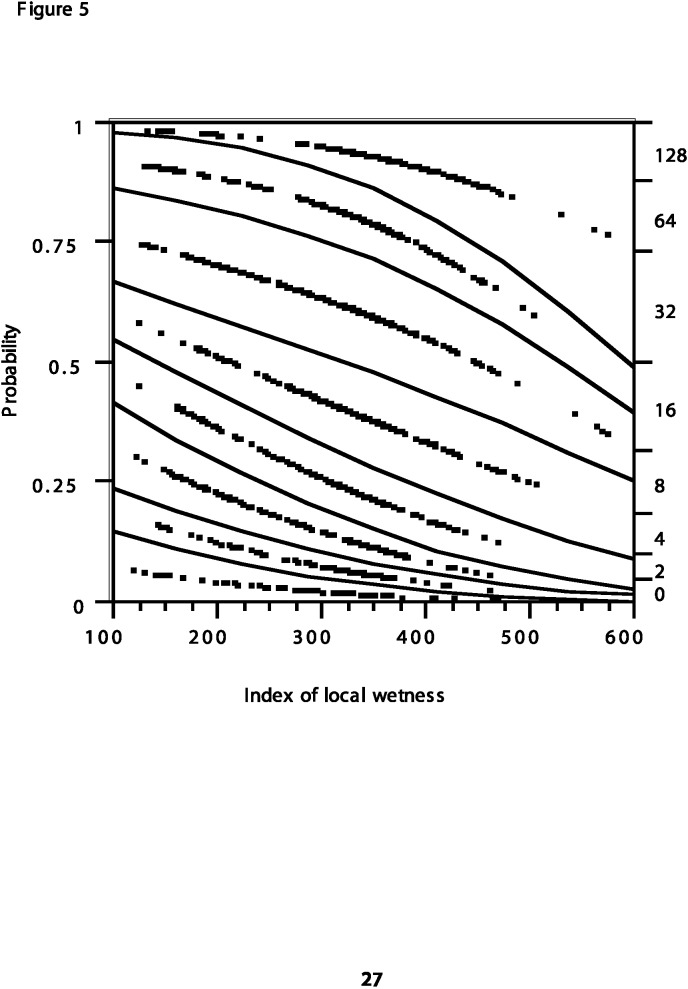
Logistic regression analysis of the complete 15-year record of Great Swamp site *Aedes vexans*. Left vertical axis provides the predicted probability that the count of mosquitoes will lie at or below a given threshold. Surface wetness (the index of local wetness [ILM]) increases from left to right. Dots between lines illustrate the distribution of ILW values for mosquito counts falling between two successive threshold line values.

**Figure 6:**
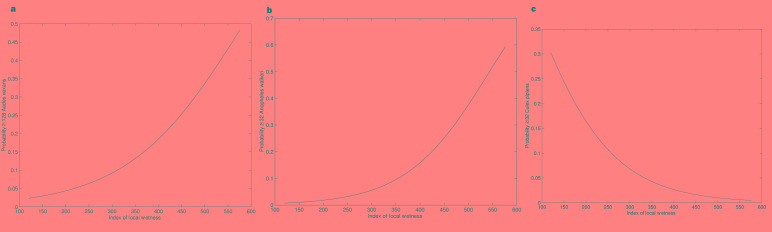
a) Mass emergence forecast, *Aedes vexans*. Mass emergence is defined as a single-day collection of ≥128 mosquitoes. The probability of a mosquito mass emergence (lagged 10 days) increases with modeled surface wetness. b) Mass emergence forecast, *Anopheles walkeri*. Based on logistic regression analysis of the 15-year record of Great Swamp site *An. walkeri.* Mass emergence is defined as a single-day collection of ≥32 *An. walkeri*. As per Figure 6a, the probability of a mosquito mass emergence (lagged 10 days) increases with increasing modeled surface wetness. c) Mass emergence forecast, *Culex pipiens*. Based on logistic regression analysis of the 15-year record of Great Swamp site *Cx. pipiens*. Mass emergence is defined as a single-day colleciton of ≥32 *Cx. pipiens*. The probability of a mosquito mass emergence (lagged 10 days) decreases with increasing modeled surface wetness.

At the Great Swamp site there is also notable year-to-year variability in the amount of variance explained by the ILW. *Ae. vexans* is significantly correlated with the ILW for only 9 of the 15 individual years; for 1986 and 1994, this association is negative. Similarly, *An. walkeri* is significantly correlated with the ILW for only 6 of 15 years examined, and for 1994, this association is also negative. While *Cx. pipiens* is negatively correlated with the ILW for the full 15-year record, only 9 of the 15 individual years have significant negative correlations; during 4 years the correlation was not statistically significant, and 2 years are significantly positively correlated (p<0.05). Multiple logistic regression was performed on all 15 years of *Ae. vexans* data at the Great Swamp site. Figure 5 presents the results of this analysis; the model fitting demonstrates that with increasing local wetness the probability of more mosquitoes 10 days later increases significantly (p<0.0001). Logistic regression was also performed singly for mass emergence levels--daily collections of ≥128 *Ae. vexans*. Model fit was significant (p<0.0001) and yielded an equation for the probability of a mass emergence of *Ae. vexans* 10 days later (Table 3). Figure 6a depicts this relation; the probability of an *Ae. vexans* mass emergence increases exponentially at the wettest surface conditions.

**Table 3:** Logistic regression equations, Great Swamp site, p <0.0001^a^

Mosquito	Probability of a mass emergence
*Aedes vexans*	p(128 *Ae. vexans*) = (1+exp(4.701 - 0.00804≥ILW))^-1^
*Anopheles walkeri*	p(≥32 *An. walkeri*) = (1+exp(6.362 - 0.0116≥ILW))^-1^
*Culex pipiens*	p(≥32 *Cx. pipiens*) = (1+exp(-0.353 + 0.00989≥ILW))^-1^

^a^Equations for the probability of mass emergence (≥128 *Ae. vexans*; ≥32 *An. walkeri*; 32 or more *Cx. pipiens*) mosquitoes) 10 days later, based on logistic regression analysis. The ILW was constructed for a depth of 0 m. Model fits are significant at p <0.0001 based on a likelihood-ratio test.

Logistic regression were also performed for the other two abundant mosquito species at the Great Swamp site. The swamp water species, *An. walkeri,* was similarly positively correlated with ILW. *Cx. pipiens* was negatively associated with ILW. Light trap collections of both of these species were fewere than collections of *Ae. vexans*; therefore, for these species, mass emergence was defined as a daily collection of ≥32 mosquitoes. Figures 6b and 6c show the probability of a mass emergence of *An. walkeri* and *Cx. pipiens*, based on these logistic regression analyses. The probability of a mass emergence of *An. walkeri* increases significantly with increasing local wetness (p<0.0001), whereas the probability of a mass emergence of *Cx. pipiens* decreases significantly with increasing local wetness (p<0.0001). Table 3 provides the model fit equations derived from these analyses.

## Discussion

The methods used here provide the basis for a dynamic mosquito prediction system. This system is designed for nonurban settings and predicts different species abundances at high spatial resolution. For the northern New Jersey watersheds we examined, the flood and swamp water species, *Ae. vexans* and *An. walkeri*, were positively associated with local modeled surface wetness 10 days before. These findings quantify the link between surface wetness and mosquito abundance and suggest that prediction of the abundance of flood and swamp water mosquitoes is practicable. In particular, the logistic regression analyses at the Great Swamp site indicate that probabilistic forecasts of localized mass emergence potential are possible. If this correspondence is shown to be robust, it could be used as a forecast tool and aid in *Ae. vexans* and *An. walkeri* control efforts in New Jersey and surrounding areas.

*Cx. pipiens* abundance was negatively associated with local modeled surface wetness 10 days prior. This species is a vector of the *West Nile virus* (*27*) and St. Louis encephalitis (*26*,*28*). The negative association between modeled surface wetness and the abundance of *Cx. pipiens* is somewhat counterintuitive; however, there is reason to believe this finding is real. *Cx. pipiens* breeds in polluted, eutrophic waters (*26*). In rural settings, such as the Great Swamp, this species makes use of swamp waters and animal waste lagoons. As these surface pools shrink in drying conditions, remaining waters grow more eutrophic. Such environmental changes favor *Cx. pipiens.* Conversely, heavy rainfall can flush the ditches and drainage channels used by *Culex* larvae. Thus, a negative association between modeled surface wetness and this species' abundance is biologically plausible in the rural Great Swamp setting. It is also consistent with studies alluding to an association between drought eutrophication and *Culex* abundance (*28*). Further corroboration is provided by collections of a less abundant species, *Uranotaenia sapphirina,* with a similar life cycle (*29*), which was also negatively associated with modeled surface wetness (data not shown). These findings suggest that it may be possible to extend the model's forecast ability to nonfloodwater species.

The logistic regression models developed here all include a 10-day lag between modeled surface wetness and subsequent mosquito abundance. Comparable associations between modeled surface wetness and subsequent mosquito abundance were usually found for lags ranging from 0 to 12 days (data not shown). This time-lag similarity is due in part to the slow variability of the water table, which fluctuates in height on time scales of weeks. The 10-day lag was chosen a priori to represent the average development time from oviposition to eclosion at the New Jersey sites for the mosquito species examined. We surmise that modeled surface wetness identifies the location of larval habitats and that the mosquitoes collected 10 days later represent newly emergent adults from these larval habitats; however, without age-grade typing of the adult collections or larval sampling data, we lack direct support for this hypothesis.

Regression analyses demonstrated that year-to-year variability exists in the association between mosquito abundances and modeled surface wetness. This finding suggests that at this time a regression model is not the best means of predicting mosquito abundance; rather, a probabilistic forecast, developed from logistic regression analysis, is more appropriate. Further study of the year-to-year variability in the association of mosquito abundance and modeled surface wetness is required.

The ability to identify potential breeding habitats accurately in space and time, for even some vector species, will enable a greater measure of control over these vectors. To be sure, other factors affect disease transmission. Host immunity, pathogen ecology, host-vector-pathogen interactions, and the effects of temperature and humidity on mosquito development rates all would require consideration for a more complete understanding and quantification of a local disease system. Nonetheless, the presence or absence of an appropriate aquatic realm, necessary for egg, larval, and pupal development, is a critical determinant of mosquito species abundances (*18*,*19*,*30*). This dependence of vector species on aquatic habitats is an invariant aspect of the mosquito life cycle and one for which the energetics can be modeled explicitly by using the fundamental, physical equations of conservation of energy and mass. Additionally, mosquito-borne diseases require mosquitoes for transmission. Thus, the dependence of mosquitoes on aquatic habitats provides both a robust link between the biological and the physical realms and a logical starting point for the dynamic modeling of mosquito-borne disease systems, provided one bears in mind the protean nature of local disease systems.

The dynamic modeling presented here has several advantages over satellite imaging approaches: 1) it models the actual aquatic environment used by the mosquitoes, not a filtered proxy; 2) it offers continuous real-time prediction of mosquitoes; 3) it resolves the whereabouts of the potential breeding habitats at a very fine scale (areas as small as 10-m cells); and 4) the model is readily coupled to global climate models, allowing additional medium- and long-range forecast of mosquito abundances.

Use of a dynamic, numerical model also allows for broad application of its methods. Adjustment of the model from region to region and climate to climate is made by accounting for differences in topography, vegetation, soil properties, and weather conditions. With these considerations the hydrology model can be transplanted to any location. All that remains to be determined is the nature of the association of modeled surface wetness and local mosquito species population numbers. Thus, dynamic hydrology models have the potential to provide public health officials with flexible methods for the assessment of current and future vector population levels, and in addition, the ability to target control efforts based on the spatial distribution of these predicted populations.

## Conclusion

We have presented methods for predicting mosquito abundance by using a dynamic hydrology model. Modeled local surface wetness was correlated with the subsequent abundance of three New Jersey mosquito species: *Ae. vexans, An. walkeri,* and *Cx. pipiens*. The sign and magnitude of the association of modeled surface wetness and species abundance appear to be a function of mosquito species biology and overall abundance. These correlations permit probabilistic forecast of mass emergences of these mosquitoes through the physical modeling of land surface hydrology.

Application of these methods across variable climate, biome, and species compositions will aid in wide-range monitoring of mosquito mass emergences through the modeling of surface wetness levels. Two types of forecast are foreseeable: 1) short term, in which catchment hydrology is modeled in real time at a fine resolution (e.g., 10-m to 30-m cells) and areas with probable high concentrations of developing larvae are identified and targeted for control; 2) long term, in which the impact of 6- to 12-month regional climate forecasts on local mosquito abundance is assessed by a coupling of climate forecast models and our hydrology model. The former system would require only the input of weather data from the nearest meteorologic station. Such monitoring could be centralized and carried out for large regions.
